# Differential effects of Nintedanib and Pirfenidone on lung alveolar epithelial cell function in ex vivo murine and human lung tissue cultures of pulmonary fibrosis

**DOI:** 10.1186/s12931-018-0876-y

**Published:** 2018-09-15

**Authors:** Mareike Lehmann, Lara Buhl, Hani N. Alsafadi, Stephan Klee, Sarah Hermann, Kathrin Mutze, Chiharu Ota, Michael Lindner, Jürgen Behr, Anne Hilgendorff, Darcy E. Wagner, Melanie Königshoff

**Affiliations:** 1 Research Unit Lung Repair and Regeneration, Helmholtz Zentrum München and University Hospital of the Ludwig Maximilians Universität, Member of the German Center for Lung Research (DZL), Munich, Germany; 2 Center for Thoracic Surgery, Asklepios Biobank for Lung Diseases, Comprehensive Pneumology Center, Asklepios Clinic Munich-Gauting, Munich, Germany; 3 Medizinische Klinik und Poliklinik V, Klinikum der Ludwig Maximilians University, Munich, Germany; 4 Department of Experimental Medical Sciences, Lung Bioengineering and Regeneration, Lund University, Lund, Sweden; 5 Wallenberg Center for Molecular Medicine, Lund University, Lund, Sweden; 6 Stem Cell Centre, Lund University, Lund, Sweden; 7 Division of Pulmonary Sciences and Critical Care Medicine, Department of Medicine, University of Colorado, AMC, Research 2, 9th Flr, 12700 East 19th Ave, Aurora, Denver, CO 80045 USA

**Keywords:** IPF, Epithelial cells, ATII, Nintedanib, Pirfenidone, ex vivo, PCLS, Lung disease

## Abstract

**Background:**

Idiopathic pulmonary fibrosis (IPF) is a fatal interstitial lung disease. Repetitive injury and reprogramming of the lung epithelium are thought to be critical drivers of disease progression, contributing to fibroblast activation, extracellular matrix remodeling, and subsequently loss of lung architecture and function. To date, Pirfenidone and Nintedanib are the only approved drugs known to decelerate disease progression, however, if and how these drugs affect lung epithelial cell function, remains largely unexplored.

**Methods:**

We treated murine and human 3D ex vivo lung tissue cultures (3D-LTCs; generated from precision cut lung slices (PCLS)) as well as primary murine alveolar epithelial type II (pmATII) cells with Pirfenidone or Nintedanib. Murine 3D-LTCs or pmATII cells were derived from the bleomycin model of fibrosis. Early fibrotic changes were induced in human 3D-LTCs by a mixture of profibrotic factors. Epithelial and mesenchymal cell function was determined by qPCR, Western blotting, Immunofluorescent staining, and ELISA.

**Results:**

Low μM concentrations of Nintedanib (1 μM) and mM concentrations of Pirfenidone (2.5 mM) reduced fibrotic gene expression including *Collagen 1a1* and *Fibronectin* in murine and human 3D-LTCs as well as pmATII cells. Notably, Nintedanib stabilized expression of distal lung epithelial cell markers, especially Surfactant Protein C in pmATII cells as well as in murine and human 3D-LTCs.

**Conclusions:**

Pirfenidone and Nintedanib exhibit distinct effects on murine and human epithelial cells, which might contribute to their anti-fibrotic action. Human 3D-LTCs represent a valuable tool to assess anti-fibrotic mechanisms of potential drugs for the treatment of IPF patients.

**Electronic supplementary material:**

The online version of this article (10.1186/s12931-018-0876-y) contains supplementary material, which is available to authorized users.

## Background

Idiopathic pulmonary fibrosis (IPF) is a fatal lung disease with a median survival of 3–5 years [1]. Repetitive lung epithelial cell injury and reprogramming along with (myo)fibroblast activation and ECM production have been proposed to be critically involved in IPF pathogenesis [1–3]. Within the distal lung, alveolar epithelial type (AT) II cells have been described to undergo significant phenotypic and functional changes upon fibrotic lung injury [4, 5], including endoplasmatic reticulum stress [6], mitochondrial dysfunction [7], or senescence [8], Moreover, these cells have been reported to release a variety of profibrotic mediators, such as Transforming growth factor (TGF)-β [9], IL-1β [10], sphingosine 1-phosphate (S1P) [11], or WNT ligands [12, 13]. Altogether, these studies support the notion that targeting the dysfunctional epithelium might be a promising therapeutic strategy for the treatment of IPF.

To date, Nintedanib and Pirfenidone are the only approved drugs known to decelerate disease progression [14, 15]. Nintedanib (BIBF-1120) is a multi-tyrosine kinase inhibitor and is known to inhibit the receptor kinases of platelet-derived growth factor (PDGF), fibroblast growth factor (FGF) and vascular endothelial growth factor (VEGF), which are all thought to play an important role in the pathogenesis of IPF [16]. Its anti-fibrotic activity has been demonstrated in multiple animal models of lung fibrosis and in in vitro assays [16–19]. In particular, Nintedanib has been shown to inhibit various fibroblast functions, such as proliferation, fibroblast to myofibroblast differentiation, and extracellular matrix (ECM) production [16–19]. However, it has been previously acknowledged that non-receptor kinases or targets not yet identified and/or effects on cells other than fibroblasts could contribute to the anti-fibrotic properties of Nintedanib [16, 20, 21]. Pirfenidone (5-methyl-1-phenyl-2-(1H)-pyridone) exhibits anti-fibrotic activity not only in the lung, but further in kidney, hepatic, and cardiac fibrosis [22–24]. Pirfenidone has anti-oxidant, anti-fibrotic and anti-inflammatory properties as shown in several in vitro and in vivo studies [22, 23].

Three-dimensional (3D) models, which allow and facilitate the study of primary lung cells in the intact lung structure and microenvironment, have recently begun to emerge. 3D-lung tissue cultures (3D-LTCs; precision cut lung slices (PCLS)) have been traditionally used for studying airway contraction, but have only recently been applied to more extended mechanistic studies ex vivo. While one major benefit is that they allow for an overall reduction of animals required for experimentation, they also permit studies directly in human tissue [25–29]. 3D-LTCs can be used to analyze human tissue-level responses to anti-fibrotic drugs, which might help to better understand the mechanisms and functional effects of drugs in human tissue and thus might better predict clinical efficacy. Recently, potential anti-fibrotic drugs have been tested on PCLS derived from IPF explants [26]. However, explanted IPF tissue is rare and represents end-stage disease at which ongoing disease mechanisms may differ significantly from earlier changes. Thus, validation of potential clinical compounds might benefit from human models, which mimic earlier stages of the disease. We have recently developed a novel model of early fibrosis-like changes in human 3D-LTCs allowing the evaluation of early pathomechanisms of IPF [25]. The effects of Nintedanib and Pirfenidone on human 3D-LTCs ex vivo, especially on lung epithelial cell function, have not yet been explored.

## Methods

### Human tissue

Tumor-free lung tissue from lung cancer resections of patients without a co-morbidity of IPF/ILD was used to generate human 3D lung tissue cultures (3D-LTCs) as previously described [25, 28]. Human tissue has been obtained from the Comprehensive Pneumology Center cohort of the BioArchive CPC-M at the University Hospital Grosshadern of the Ludwig Maximilian University (Munich, Germany) and by the Asklepios Biobank of Lung Diseases (Gauting, Germany). Participants provided written informed consent to participate in this study, in accordance with approval by the local ethics committee of the LMU, Germany (Project 333-10, 455-12). Tumor-free tissue from lung cancer resection surgeries of patients without an IPF/ILD diagnosis was used comprising the following: 2 patients (1 male 62 years, 1 female, 80 years) with squamous cell carcinoma and 1 patient with a carcinoid tumor of the lung (male, 48 years). No information on smoking history was available for these patients. Absence of IPF/ILD was confirmed by CT and pathology.

### Animal experiment

Pathogen-free female C57BL/6 mice (6–8 wk. old) purchased from Charles River were used in all studies. The mice were housed in rooms with constant humidity and temperature with 12 h light cycles and had free access to water and food. All experiments were performed in accordance with the guidelines of the ethics committee of the Helmholtz Zentrum Munich (Germany) and approved by the regional council of Upper Bavaria Germany (Project 55.2–1-54-2532-88-12). For the induction of experimental fibrosis, a single dose of Bleomycin (2 U/kg body weight; Bleomycin sulfate, Almirall, Spain, dissolved in 50 μl sterile PBS) was intratracheally administered using the Micro-Sprayer Aerosolizer (Penn-Century, Wyndmoor, PA). Control mice received 50 μl PBS. Fourteen days after instillation the mice were sacrificed and the lungs were harvested for the generation of pmATII cells or 3D-LTCs.

### Cell culture

The pmATII cells were isolated from mice as previously described [8]. The pmATII cells were seeded in 12 well-tissue culture plates and cultured in DMEM-F12 supplemented with 10% FCS, 2 mM 1-glutamine, 1% penicillin/streptomycin, 3.6 mg/ml glucose and 10 mM HEPES for 24 h to allow attachment. Cells were then cultured for 12 h in fresh 0.1% FCS containing medium. Subsequently, cells were pre-treated with Nintedanib (1 μM) (Selleck, Houston, TX) or Pirfenidone (500 μM) (Selleck, Houston, TX) or respective DMSO control for 48 h.

### Human and murine 3D-lung tissue culture (3D-LTCs) ex vivo

Human and murine 3D-LTCs and 4 mm-punches thereof were generated as previously described [25, 28]. The amount of slices generated from one mouse lung varied between 18 and 25 slices, determining the amount of further downstream analysis. The 3D-LTCs were cultured in DMEM-F12 containing 0.1% FCS and 1% penicillin/streptomycin. 3D-LTCs obtained from mice subjected to PBS or Bleomycin were stimulated either with Nintedanib (0.1 μM, 1 μM, 10 μM) or Pirfenidone (100 μM, 500 μM, 2.5 mM) for 48 h.

Human 3D-LTCs were treated with a fibrosis cocktail (FC) consisting of TGF-β, Platelet-derived growth factor (PDGF)-AB, tumor necrosis factor (TNF)-α and Lysophosphatidic acid (LPA) [25]. Briefly, slices and 4-mm biopsy punches were treated with FC or control cocktail (CC) for 48 h followed by the co-treatment of Nintedanib (1 μM) or Pirfenidone (500 μM) with FC or CC for 72 h (Fig. 4a). Supernatants from punches were pooled for each condition and stored at − 80 °C for further analysis. A WST-1 assay was performed as previously described [25]. Punches were fixed with 4% paraformaldehyde (PFA) for 30 min and subsequently washed with 1 X DPBS. Slices were snap-frozen in liquid nitrogen and stored at − 80 °C.

### Immunofluorescence (IF)

IF was performed as previously described [25, 28]. Briefly, 3D-LTCs were fixed with acetone/methanol (AppliChem, Germany) for 20 min or as indicated otherwise, blocked with 5% bovine serum albumin (Sigma Aldrich, UK) in PBS for 1 h at RT and subsequently incubated in primary antibody diluted in PBS containing 0.1% BSA over night at 4 °C followed by secondary antibody incubation for 2 h at room temperature and DAPI (Roche, Switzerland) stain for 10 min. Images were obtained using LSM710 confocal microscope (Zeiss, Germany). 3D images were reconstructed using IMARIS× 64 (v9.0; Bitplane, Zurich, Switzerland).

### RNA isolation and quantitative (q)RT-PCR

Two to three 3D-LTCs were pooled, snap frozen in liquid nitrogen and homogenized using a tissue lyser as previously described [25, 28]. Peqlab Total RNA Kit (Peqlab, Germany) was used for total RNA isolation from cells and murine 3D-LTCs with modifications of the manufacturer’s instructions. Total RNA from human 3D-LTCs was extracted using the RNeasy Fibrous tissue kit (Quiagen, Germany) with Peqlab DNA removing columns prior to RNA binding. The RNA concentration and quality was assessed using NanoDrop spectrophotometer (Thermo Fisher Scientific, Germany). qRT-PCR was performed using SYBR Green (Roche, Switzerland) and the LC480 Light Cycler (Roche, Switzerland). *HPRT* for mouse and human was used as a reference gene in all qRT-PCR reactions. The relative gene expression is defined as ΔCp value (ΔCp = (Cp Hprt)-(Cp gene of interest)). Logfold change as ΔΔCp = ΔCp (treatment) -ΔCp(Control). The following primer sequences were used: *mFn1-F*,5’-GGTGTAGCACAACTTCCAATTACG-3′; *mFn1-R*, 5’-GGAATTTCCGCCTCGAGTCT-3′; *mCol1a1-F*, 5’-CCAAGAAGACATCCCTGAAGTCA-3′, *mCol1a1-R*, 5’-TGCACGTCATCGCACACA-3′; *mSftpc-F*, 5’-AGCAAAGAGGTCCTGATGGA-3′; *mSftpc-R*, 5’-GAGCAGAGCCCCTACAATCA-3′; *mT1a-F*, 5’-ACAGGTGCTACTGGAGGGCTT-3′; *mT1a-R*, 5’-TCCTCTAAGGGAGGCTTCGTC-3′; *mNkx2.1-F*, 5′- AGGACACCATGCGGAACAG-3′; *mNkx2.1-R*, 5’-CCATGCCGCTCATATTCATGC-3′; *mHopx-F*, 5’-TCTCCATCCTTAGTCAGACGC-3′; *mHopx-R*, 5’-GGGTGCTTGTTGACCTTGTT-3′; *huCDH1-F*, 5’-ATACACTCTCTTCTCTCACGCTGTGT-3′; *huCDH1-R*, 5’-CATTCTGATCGGTTACCGTGATC-3′; *huZO1-F*, 5’-TCTGAGCCTGTAAGAGAGGAC-3′; *huZO1-R*, 5’-GCTTCTGCTTTCTGTTGAGAGG-3′; *huSFTPC-F*, 5’-GCCCAGTGCACCTGAAACGC-3′; *huSFTPC-R*, 5’-TCTCCAGAACCATCTCCGTGTGT-3′; *hNKX2.1-F*, 5’-AGCACACGACTCCGTTCTC-3′; *hNKX2.1-R*, 5’-GCCCACTTTCTTGTAGCTTTCC-3′.

### Immunoblotting

Pulverized 3D-LTCs were lysed in T-PER lysis buffer (Thermo Fisher Scientific, Germany) containing proteinase and phosphatase inhibitors (Roche, Switzerland). Protein concentration was assessed using the BCA assay (Thermo Fisher Scientific, Germany) according to the manufacturer’s instructions. 15 μg of total protein was separated on SDS-polyacrylamide gels and transferred to PVDF membranes (Biorad, USA). The membranes were blocked in 5% nonfat dry milk (Applichem, Germany) and incubated with the primary antibody (at 4 °C overnight followed by 1 h at RT). Subsequently the blots were incubated with respective secondary, HRP-conjugated, antibody (GE-Healthcare) for 1 h, washed and visualized using chemiluminescence reagents (Pierce ECL, Thermo Fisher Scientific, Germany) with the ChemiDocTMXRS+ system. Analysis of secreted collagen was performed by concentrating 200 μl of supernatant from the same number of 4-mm punches generated from 3D-LTCs in each group using Nanosep 10 K OMEGA columns (Pall Corporation, MI, USA) followed by dilution in 60 μl lysis buffer and as previously described [25].

### Antibodies

Primary antibodies for immunoblotting and immunofluorescence were as follows: anti proSP-C, ab40879 (Abcam, UK; WB 1:1000, IF 1:200); anti Collagen1, 600–401-103 (Rockland, USA; WB 1:1000, IF 1:200)); anti fibronectin ((H-300) (sc-9068, SantaCruz, Heidelberg, Germany; IF 1:200); anti E-cad (610181, BD, Franklin Lakes, NJ, USA, IF 1:200); anti aSMA (ab5694, Abcam, UK, IF 1:100); anti β-actin, A3854 (Sigma Aldrich, UK; WB 1:25000). Alexafluor conjugated (488, 555 or 647) anti-mouse and anti-rabbit secondary antibodies (Thermo Fisher Scientific, Germany) were used for IF.

### ELISA

Supernatants were taken from cultures of murine and human 3D-LTCs. Secreted protein content was determined by enzyme-linked immunosorbent assay (ELISA) according to the manufacturer’s instructions (m/hWISP1 – DY1627, R&D, MN, USA; mSP-C - CSB-E12639m, Cusabio, MD, USA; hSP-C - CSB-E10135h, Cusabio, MD, USA).

### Statistical analysis

All data is presented as mean ± SEM and was generated using GraphPad Prism 5. Statistical significance was evaluated with either Wilcoxon signed-rank test, Mann-Whitney U test or repeated-measures one-way ANOVA followed by Newmann-Keuls multiple comparison test or two-way ANOVA followed by Sidak’s multiple comparison test or significance of log-fold change was evaluated with one-sample t-tests in comparison to a hypothetical value of 0. Differences were considered to be statistically significant when *P* < 0.05.

## Results

**Fig. 1:**
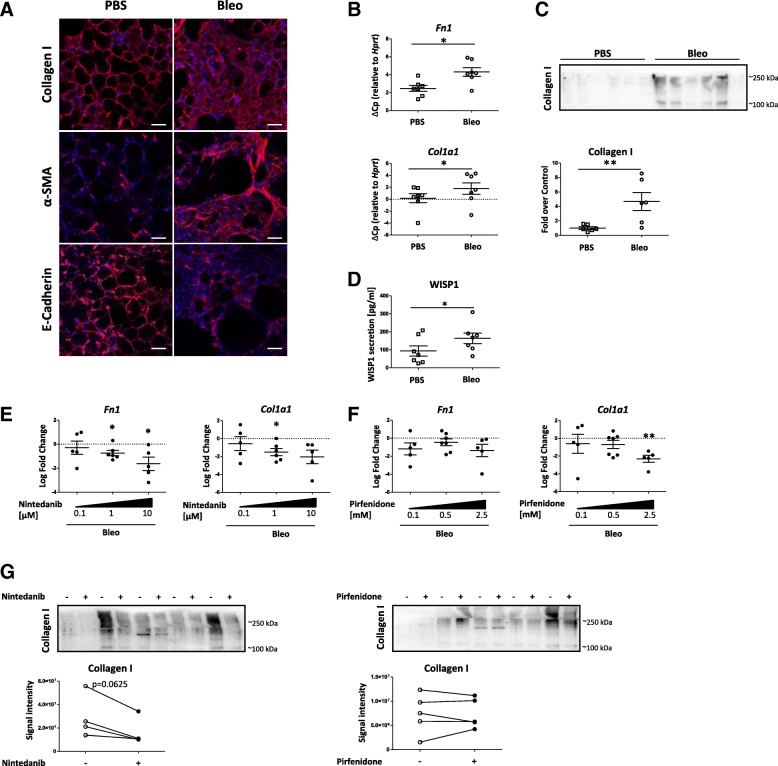
Effect of ex vivo treatment with Pirfenidone and Nintedanib on the fibrotic phenotype of 3D-LTCs. **a** Representative immunofluorescence analysis of Collagen I, α-SMA and E-Cadherin in control (PBS) and fibrotic (Bleo) 3D-LTCs after 48 h in culture. Scale bar represents 50 μm. **b** Gene expression analysis by qPCR of fibrotic marker *Fn1* and *Col1a1* in control and fibrotic 3D-LTCs after 48 h in culture. ΔCp relative to *Hprt* is presented as mean ± SEM, *n* = 7. Means were compared using Wilcoxon matched pairs test. **c** Collagen I secretion of control and fibrotic 3D-LTCs was determined by WB and normalized to supernatant volume. *n* = 6. Means were compared using Mann-Whitney test. **d** WISP1 secretion of control and fibrotic 3D-LTCs was measured by ELISA. n = 7. Significance was assessed using Wilcoxon matched pairs test. **e**, **f** Fibrotic 3D-LTCs were cultured for 48 h in the presence of anti-fibrotic drugs (**e**) Nintedanib (0.1 μM, 1 μM, 10 μM) (**f**) and Pirfenidone (100 μM, 500 μM, 2.5 mM). Gene expression analysis by qPCR of fibrotic marker *Fn1* and *Col1a1*. Log fold change is presented as mean ± SEM, *n* = 5–7. Means were compared to respective DMSO control using one-sample t-tests in comparison to a hypothetical value of 0. **g** Collagen I secretion of fibrotic 3D-LTCs treated with Nintedanib (1 μM) and Pirfenidone (500 μM) for 48 h was determined by WB and normalized to supernatant volume. *n* = 5. Significance was assessed using Wilcoxon matched pairs test. Significance: *p < 0.05, **p < 0.01

Nintedanib and Pirfenidone have both been shown to exhibit anti-fibrotic capacities in animal models of lung fibrosis in vivo. As our ultimate goal was to test the effect of the compounds in an ex vivo model of human pulmonary fibrosis, we sought to establish the feasibility of this approach by using murine ex vivo models. Mice were subjected to intratracheal bleomycin administration and 3D-LTCs were generated after 14 days of in vivo fibrosis development (Additional file 1: Figure S1), representing a time point with established fibrosis where preclinical testing of potential anti-fibrotic drugs has been recommended [30]. We first established baseline characteristics of freshly generated healthy and fibrotic murine 3D-LTCs and found that 3D-LTCs derived from fibrotic mice maintained the fibrotic lung structure ex vivo, as indicated by dense staining of collagen and increased alpha-SMA, accompanied by decreases in E-cadherin expression, thus demonstrating that 3D-LTC generation does not significantly affect baseline differences (Fig. 1a). To further characterize the fibrotic phenotype of 3D-LTCs in culture, fibrotic markers were analyzed after 48 h. As shown in Fig. 1b, the expression of the mesenchymal marker genes *Fibronectin 1 (Fn1)* and *Collagen (Col) 1a1* were both significantly upregulated compared to the PBS control. In line with this, the secretion of total collagen analysed by Western Blotting was significantly increased (Fig. 1c). Furthermore, we found that the secretion of Wnt1-inducible signaling protein (WISP) 1, a protein increased in the distal pulmonary epithelium of fibrotic mice and in human fibrosis, was significantly upregulated at 48 h (Fig. 1d) [8, 13].

Given that the fibrotic phenotype is maintained in ex vivo 3D-LTCs, we next analyzed the therapeutic effects of Pirfenidone and Nintedanib (Fig. 1e, f). We observed dose-dependent effects for both compounds. Nintedanib significantly downregulated mRNA levels of *Fn1* and *Col1a1* at 1 μM (− 0.94 ± 0.25 and − 1.51 ± 0.99, respectively; log fold change compared to control), while Pirfenidone significantly downregulated *Col1a1* at 2.5 mM (− 1.36 ± 1.39 and − 1.95 ± 1.07, respectively; log fold change compared to control). Furthermore, the secretion of collagen, as analyzed by Western blotting, showed a trend towards downregulation upon Nintedanib treatment but was not changed by Pirfenidone treatment in fibrotic 3D-LTCs (0.61 ± 0.16 and 1.28 ± 0.82 for Nintedanib and Pirfenidone, respectively; fold change upon treatment) (Fig. 1g). Both drugs exhibited similar effects on fibrotic gene expression in 3D-LTCs derived from PBS treated mice, except no significant effect on Collagen 1 secretion (Additional file 2: Figure S2A-C). Overall, these data confirm the previous reported anti-fibrotic effects of Pirfenidone and Nintedanib in experimental lung fibrosis models in vivo in an ex vivo tissue culture model and demonstrate that 3D-LTCs can be applied to further investigate the effect of both drugs on cellular phenotypes and function. Hereafter, we used concentrations of 1 μM Nintedanib and 500 μM Pirfenidone as these concentrations have been widely used and recommended in in vitro studies [18] and showed anti-fibrotic activity in our ex vivo model (statistically significant for Nintedanib; trend for Pirfenidone).

**Fig. 2:**
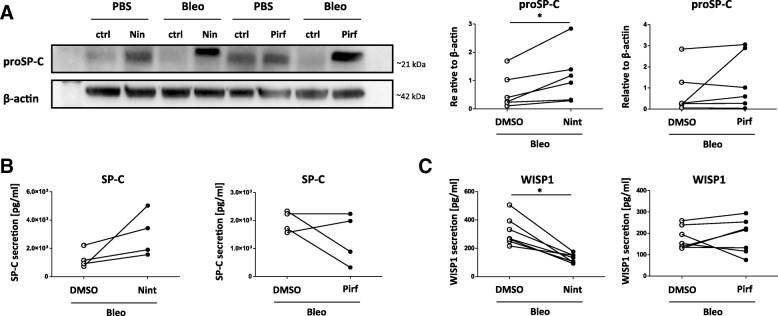
Effect of ex vivo treatment with Pirfenidone and Nintedanib on lung epithelial cell marker in fibrotic 3D-LTCs. **a-c** Fibrotic 3D-LTCs were cultured for 48 h in the presence of anti-fibrotic drugs Nintedanib (1 μM) and Pirfenidone (500 μM). **a** proSP-C expression was assessed by Western blot. β-Actin was used as loading control. Quantification of proSP-C Western blot, n = 6. Data was normalized to β-Actin. **b**, **c** SP-C and WISP1 secretion of 3D-LTCs was determined by ELISA. *n* = 4–7. Significance was assessed using Wilcoxon matched pairs test. Significance: *p < 0.05

While the anti-fibrotic effects of both drugs have been predominantly studied in fibroblasts [16–19, 22–24], there is little knowledge about the effects of Nintedanib and Pirfenidone on the lung epithelium. We first assessed changes of the functional ATII cell marker pro surfactant protein C (SP-C) and found that Nintedanib increased proSP-C protein expression (Fig. 2a) and affected SP-C secretion in fibrotic 3D-LTCs (Fig. 2b and Additional file 3: Figure S3A and B). In order to determine if Nintedanib treatment was also able to suppress epithelial-derived pro-fibrotic mediator expression, we examined secretion of WISP1, which was attenuated in both fibrotic and normal 3D-LTCs by Nintedanib as assessed by ELISA (Fig. 2c and Additional file: 3 Figure S3C). In contrast, Pirfenidone did not consistently induce SP-C secretion or proSP-C expression (Fig. 2a and b), nor affected WISP1 secretion in fibrotic 3D-LTCs (Fig. 2c). In order to rule out that higher Pirfenidone concentrations could more consistently affect epithelial cells, we tested a concentration of 2.5 mM Pirfenidone, which did not show any significant effect on *Sftpc* gene expression (Additional file: 3 Figure S3D).

**Fig. 3:**
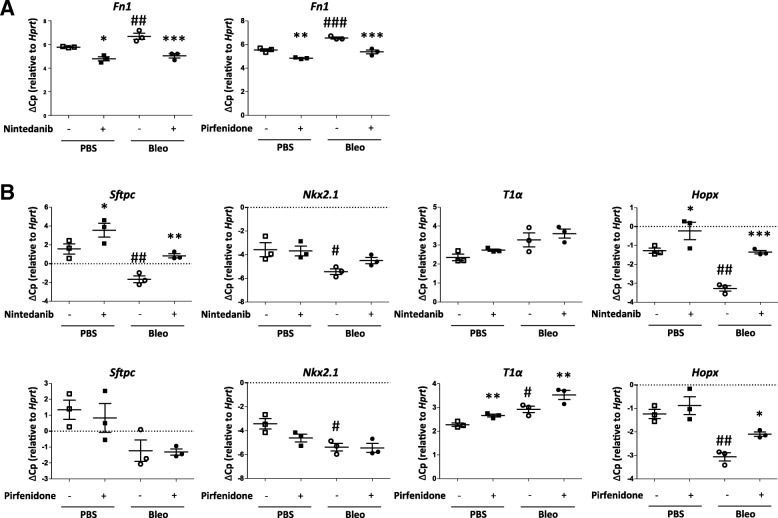
Effect of in vitro treatment with Pirfenidone and Nintedanib on primary mouse (pm)ATII cells. **a**, **b** At day 14 after Bleomycin instillation, mice were sacrificed and control (PBS) and fibrotic (Bleo) pmATII cells were harvested. The pmATII cells were cultured in the presence of Nintedanib (1 μM) and Pirfenidone (500 μM) for 48 h. **a** Gene expression analysis by qPCR of fibrotic marker *Fn1* in pmATII cells. ΔCp *relative to Hprt* is presented as mean ± SEM, *n* = 3. Means were compared using repeated-measures one-way ANOVA followed by Newmann-Keuls post test. **b** Gene expression analysis by qPCR of epithelial cell markers *Sftpc*, *Nkx2.1*, *T1α*, *Hopx*. ΔCp *relative to Hprt* is presented as mean ± SEM, n = 3. Means were compared using repeated-measures one-way ANOVA followed by Newmann-Keuls post test. Significance: *p < 0.05, **p < 0.01, ***p < 0.001 (DMSO vs Pirfenidone/Nintedanib). Significance: #*p* < 0.05, ##*p* < 0.01, ###*p* < 0.001 (PBS vs Bleo)

Next, we aimed to validate these findings in fibrotic pmATII cells (Fig. 3). As described previously [8], the expression of *Fn1* was significantly increased in cultured pmATII cells derived from bleomycin-instilled mice compared to the PBS control (PBS vs Bleo: 5.78 ± 0.04 vs 6.69 ± 0.37; relative gene expression) (Fig. 3a). We then investigated gene expression changes of phenotypic and functional ATII cell markers, including surfactant protein C (*Sftpc)*, *Nkx2.1* (thyroid transcription factor 1 (TTF-1)), ATI cell marker (T1a (Podoplanin)), and a marker of a putative bi-potent progenitor population for ATI and ATII cells *(homeodomain only protein X* (*Hopx*)). Notably, fibrotic pmATII cells exhibited reduced expression of *Sftpc* as well as *Nkx2.1* and *Hopx* expression, whereas *T1α* (Podoplanin*;* a marker for ATI cells;) was increased (Fig. 3b), likely representing attempted repair by transdifferentiation of ATII to ATI cells [31, 32]. *Fn1* was significantly downregulated by both Nintedanib and Pirfenidone in healthy and fibrotic pmATII cells as assessed by qPCR (Fig. 3a). In line with our previous observations in 3D-LTCs, treatment with Nintedanib restored the expression of *Sftpc, Nkx2.1* and *Hopx* to the level of the PBS control (Fig. 3b). Pirfenidone treatment increased both *Hopx* and *T1α* but did not affect ATII cell markers *Sftpc* and *Nkx2.1* expression (Fig. 3b).

**Fig. 4:**
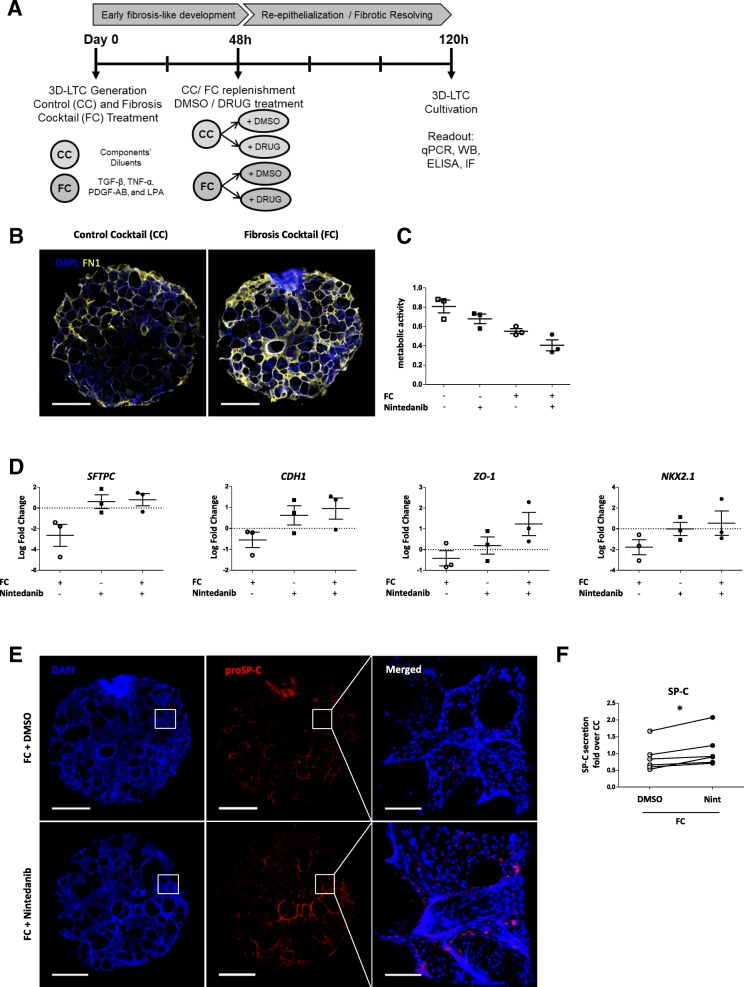
Ex vivo treatment with Nintedanib stimulates alveolar epithelial marker expression in the human 3D-LTCs model of early pulmonary fibrosis**. a** Schematic of treatment with fibrotic cocktail (FC) or control cocktail (CC) and Nintedanib (Nint) and downstream analysis. 3D-LTCs were generated and treated with FC or CC for 48 h before FC or CC treatment was replenished and Nintedanib or control treatment was added. Treatment was stopped after 120 h and downstream experiments were performed. **b** Representative Immunofluorescence of punches treated with CC or FC for 120 h and stained for Fibronectin. Scale bars represent 1 mm. **c**-**f** Punches were treated with CC/FC and Nintedanib (1 μM) as indicated in (**a**). **c** Metabolic activity of punches 120 h after treatment with CC/FC and co-treatment with Nintedanib. *N* = 3. Significance was assessed by two-way ANOVA followed by Sidak’s multiple comparisons test. **d** Gene expression analysis by qPCR of epithelial cell marker *SFTPC*, *NKX2.1*, *CDH-1, ZO-1*. Log fold change is presented as mean ± SEM, *N* = 3. Means were compared to respective DMSO control using one-sample t-tests in comparison to a hypothetical value of 0. **e** Representative Immunofluorescence of punches treated with FC and Nintedanib for proSP-C. Scale bars represent 140um. **f** SP-C secretion of punches 120 h after treatment with CC/FC and co-treatment with Nintedanib was measured by ELISA. Values shown are normalized to CC treatment. Significance was assessed using Wilcoxon matched pairs test. *N* = 6. Significance: *p < 0.05

Most mechanistic studies regarding Nintedanib or Pirfenidone have been performed on isolated cells or in mice. We thus next investigated the effects of Nintedanib (1 μM) and Pirfenidone (500 μM) in human 3D-LTCs and particularly focused on their effects on distal lung epithelial cell markers (Fig. 4). We recently developed a model that induces early fibrotic like changes in human 3D-LTCs using a combination of four growth factors and signaling molecules known to be elevated in fibrosis (FC; TGF-β, PDGF-AB, TNF-α and LPA), (Additional file 1: Figure S1, Fig. 4a) [25]. Induction of early fibrosis-like changes in our cohort by FC was confirmed by fibronectin deposition as evaluated by immunofluorescent staining (Fig. 4b). Importantly, treatment of FC-treated 3D-LTCs with Nintedanib or Pirfenidone did not alter metabolic activity as measured by WST-1 activity, suggesting that overall cell survival was not affected (Fig. 4c, Additional file 4: Figure S4A). In line with our previous findings, Nintedanib treatment restored epithelial gene expression (Fig. 4d) as well as proSP-C protein expression (Fig. 4e) and SP-C secretion (Fig. 4f). In contrast, Pirfenidone did not affect epithelial cells in this model (Additional file 4: Figure S4B and C). Overall, Nintedanib upregulated ATII cell marker expression in both murine and human 3D-LTCs and pmATII cells, whereas Pirfenidone did not significantly affect the expression of the analyzed ATII cell markers in our study.

## Discussion

IPF is a devastating interstitial lung disease, which progressively leads to lung destruction and loss of lung function. Therapeutic options are limited, with two approved drugs, Pirfenidone and Nintedanib, which decelerate the loss of lung function compared to placebo-treated patients [14, 15]. To date, there have been no studies on living human lung tissue and thus we have limited insights into their mode of action in humans. A deeper and more comprehensive understanding of the potential human target cells and target proteins of Nintedanib and Pirfenidone may help in selecting which patients will respond better to which treatment and may further allow for development of new drugs which are designed to be more specific while at the same time reducing off-target and side effects. 3D-LTCs are emerging as a potential novel model, which can be used to bridge pre-clinical data to the clinical data. Here, we report for the first time anti-fibrotic activities of Pirfenidone and Nintedanib in murine and human fibrotic 3D-LTCs ex vivo and explored the effect of Nintedanib and Pirfenidone on alveolar epithelial cells. Notably, we provide evidence that both drugs exhibit different effects on alveolar epithelial cell behavior and function, with Nintedanib increasing in particular ATII cell markers.

Repetitive injury to the alveolar epithelium represents a major pathomechanism in the development of IPF [2]. Aberrant responses of ATII cells have been shown to contribute to impaired repair and regeneration processes. However, development of novel therapeutic agents has largely focused on targeting increased ECM production and fibroblast accumulation. Several recent studies underscore the suitability and effectiveness of targeting aberrant epithelial cell responses in pulmonary fibrosis [8, 13, 33–35]. Most mechanistic studies about Pirfenidone and Nintedanib have thus far been performed on fibroblasts [16–19, 21, 36, 37]. To date, we know very little about how Nintedanib and Pirfenidone influence other cell types in the lung in their natural 3D composition, including epithelial cells. There is previous evidence that Nintedanib decreases the proliferation of vascular endothelial cells [20, 38]. Pirfenidone was found to inhibit the shedding of microparticles [39], and Nintedanib increased SP-D expression in a human lung epithelial cell line [40], however, these studies used supraphysiologial concentrations of the drugs and are thus of concern for interpreting these results in the context of how these drugs might function in patients [41]. Two recent reports suggest an inhibition of EMT by Nintedanib in ovarian cancer cells along with increased E-cadherin levels [42, 43]. In line with these observations, we consistently found that Nintedanib improves functional alveolar epithelial cell markers, such as SP-C, in our study. These data suggest that restoring normal alveolar epithelial cell function might contribute to the anti-fibrotic effects of Nintedanib. Future studies using relevant in vivo models will be required to further investigate and prove a potential causal relationship.

The use of Nintedanib and Pirfenidone in in vitro experiments has been reported in a wide range of concentrations in the literature (up to 10 mM for Pirfenidone and 5 μM for Nintedanib) [40, 44, 45]. After initial testing of different concentrations in our 3D-LTC model, we opted to use the drugs at concentrations that have been widely used and recommended in in vitro studies (Nintedanib: 1 μM and Pirfenidone: 500 μM) [18]. However, the concentrations we used slightly exceed concentrations measured in the patients’ plasma treated with either drug (around 100 nM for Nintedanib and around 100 μM for Pirfenidone) [41, 46], and might thus be representative of local concentrations of the drugs in the lung. The availability of the drug in the 3D-LTCs might differ as compared to in vitro or in vivo experiments due to methodological reasons, nonetheless, our ability to detect effects of the drug in our setup are encouraging as to the applicability of the model for pre-clinical testing of small molecules. Notably, while Nintedanib had an effect on several epithelial cell marker, which have been associated with distinct phenotypes, we did not observe a consistent effect of Pirfenidone across this same panel of lung epithelial cell markers in our studies. This might be due to limited concentrations and/or time points that we were able to analyze, however, these data further could indicate that individual patients exhibit different responses to Pirfenidone, which should be further determined in larger cohorts.

While the ex vivo system includes many different cell types and direct effects on non-epithelial cell types could contribute to the observed changes in epithelial cells, the effects we observed in pmATII cells suggests that the increase in epithelial cell markers could be mediated by a direct effect on these cells. Further studies aiming to decipher the mechanism of how Nintedanib regulates epithelial cell markers will be important to potentially identify which patients may respond to which treatment. One possible mechanism that might partially explain our findings is that Nintedanib increased the expression of the transcription factor Nkx2.1 in isolated pmATII cells and in murine and human 3D-LTCs. Nkx2.1 is a critical transcription factor in lung endoderm specification and is known to control *Sftpc* gene expression in adult ATII cells [47]. As we further observed increased expression of other adult distal lung epithelial markers not regulated by Nkx2.1, such as *Hopx* and *Pdpn,* other transcriptional regulators such as Retinoid X receptors (RXR) are likely involved [48].

3D-LTCs from rodents or human lung tissue have gained increasing attention as disease models and for preclinical validation of new therapeutics or in personalized medicine approaches [25–28, 49]. 3D-LTCs spatially retain the majority of the cellular diversity of the native lung and allow for the analysis of tissue-level responses to anti-fibrotic drugs in living tissue ex vivo. Moreover, murine 3D-LTCs can be applied to extend mechanistic studies, while reducing overall animal experimentation [30]. Nevertheless, 3D-LTCs have certain limitations, for example they lack appropriate control of air liquid interfaces, are not undergoing cyclic stretch and there is no ability to recruit cells. This system does, however, offer the unique opportunity to test drugs in the native human lung microenvironment. The evaluation of approved drugs, such as Nintedanib or Pirfenidone in these emerging ex vivo models might pave the way for further drug screening and validation for IPF. Fibrotic 3D-LTCs have been previously shown to maintain their fibrotic phenotype in culture for up to 7 days [27]. Similarly, we found that murine fibrotic slices derived from the bleomycin model maintained elevated expression of fibrotic markers after 48 h of culture. Here, we further show that functional alveolar epithelial cell markers such as SP-C were consistently downregulated over culture thus indicating that alveolar injury upon bleomycin exposure [50], is maintained ex vivo. These data underline that murine 3D-LTCs can be applied as appropriate models to study anti-fibrotic drugs in this timeframe, including their effects on epithelial cells. Recent studies have taken advantage of 3D lung slice cultures ex vivo. Tatler et al. demonstrated that caffeine reduced established fibrosis in 3D-LTCs from Bleomycin-instilled mice [27], and IPF slices were used to determine the potential therapeutic effect of a PI3K inhibitor [26]. Both studies highlight the suitability of both mouse and human 3D-LTCs in the preclinical testing of anti-fibrotic drugs. However, the effects of the approved drugs Pirfenidone and Nintedanib on fibrotic 3D-LTCs have not been explored yet. Pirfenidone has been shown to reduce Collagen1 expression in a rat slice model of liver fibrosis [51]. Similarly, in our bleomycin-induced fibrotic murine lung slices, we found that Pirfenidone decreased fibrotic markers. However, to date, there have been no ex vivo studies in any tissue utilizing Nintedanib. Consistent with in vivo experiments showing reduced collagen deposition upon treatment [19], we show that Nintedanib reduces established fibrotic markers in lung slices from bleomycin-treated animals. Recent reports describe the safety and tolerability of a combination treatment with Pirfenidone and Nintedanib thus suggesting a future treatment option for IPF patients [52]. Our study further identifies differences in the mode of action of these two drugs and further analysis on combined treatment in ex vivo models will be helpful to gain further insight into the potential rational for combining Nintedanib and Pirfenidone as well as other potential novel drugs.

While the use of human tissue for the generation of 3D-LTCs represents a unique possibility for a human disease model [25, 26, 28, 49], one major limitation is that access to IPF lung explants is limited and those available for research typically represent only end-stage disease. To test the effects of Nintedanib and Pirfenidone in human 3D-LTCs upon fibrotic remodeling, we treated human 3D-LTCs with a combination of different pro-fibrotic growth factors, pro-inflammatory cytokines and signaling molecules that induce early fibrosis-like features in human 3D-LTCs [25]. While the FC model demonstrates robust changes in ECM remodeling, it is important to highlight that further development and improvement of ex vivo 3D-LTC models to induce and reflect even more IPF-like fibrotic responses, for example with respect to other environmental challenges, aging, or genetic susceptibility, will be essential to allow the full exploitation of these models to discover and confirm novel drugs for the treatment of IPF.

## Conclusion

In summary, we report that Nintedanib but not Pirfenidone treatment positively affects a wide panel of phenotypic markers of different alveolar epithelial cell types in vitro and ex vivo, potentially contributing to its anti-fibrotic activity. In addition, we demonstrate that Nintedanib exhibits anti-fibrotic activity in an ex vivo model of IPF using human tissue, further validating this model for use in preclinical studies.

## Additional files

Additional file 1: **Figure S1.** Generation of mouse and human 3D-LTCs. Mouse lungs were harvested from PBS or Bleomycin treated mice or cancer resections from human patients were collected and filled with 2 or 3% low melting agarose, respectively. 300 or 500 μm thin 3D-LTCs were generated and cultured as indicated in each experiment. (PDF 90 kb)

Additional file 2: **Figure S2.** Effect of Pirfenidone and Nintedanib on fibrotic marker in healthy 3D-LTCs ex vivo. Mice were instilled with PBS sacrificed at day 14. 3D-LTCs were generated and cultured for 48h. (A,B) The non-fibrotic 3D-LTCs were cultured for 48h in the presence of anti-fibrotic drugs (A) Nintedanib (0.1μM, 1μM, 10μM) (B) and Pirfenidone (100μM, 500μM, 2.5mM). Gene expression analysis by qPCR of fibrotic marker Fn1 and Col1a1. ΔΔCt relative to Hprt and respective DMSO control is presented as mean ± SEM, *n* = 5-7. Means were compared to respective DMSO control using one-sample t-tests in comparison to a hypothetical value of 0. (C) Collagen I secretion of nonfibrotic 3D-LTCs treated with Nintedanib (1μM) and Pirfenidone (500μM) was determined by WB and normalized to supernatant volume. *n* = 5. Significance: *p < 0.05, **p < 0.01. (PDF 166 kb)

Additional file 3: **Figure S3.** Effect of Pirfenidone and Nintedanib on epithelial cell marker in non-fibrotic 3D-LTCs ex vivo (A-C) Fibrotic and non-fibrotic 3D-LTCs were cultured for 48h in the presence of anti-fibrotic drugs Nintedanib (1μM) and Pirfenidone (500μM). (A) proSP-C expression was assessed by Western blot. β-Actin was used as loading control. Quantification of proSP-C Western blot. Data was normalized to β-Actin. Significance was assessed using Wilcoxon matched pairs test, *n* = 6. (B, C) SP-C and WISP 1 secretion of non-fibrotic 3D-LTCs was determined by ELISA. Significance was assessed using Wilcoxon matched pairs test, *n* = 4-7. (D) Fibrotic and non-fibrotic 3D-LTCs were cultured for 48h in the presence of 2.5mM Pirfenidone. Gene expression analysis by qPCR of ATII marker Sftpc. ΔCt is presented as mean ± SEM, *n* = 5. Means were compared using repeated-measures one-way ANOVA followed by Newmann-Keuls post test. Significance: *p < 0.05. (PDF 748 kb)

Additional file 4: **Figure S4.** Effect of Pirfenidone on ex vivo human PCLS model of IPF. (A-C) Punches were treated with CC/FC and Pirfenidone (500 μM) as indicated in (Fig. 4a). (A) Metabolic activity of punches 120h after treatment with CC/FC and cotreatment with Pirfenidone. *N* = 3. Significance was assessed by two-way ANOVA followed by Sidak’s multiple comparisons test. (B) Gene expression analysis by qPCR of epithelial cell marker SFTPC, NKX2.1, CDH-1, ZO-1. ΔΔCt is presented as mean ± SEM, *n* = 3. Means were compared to respective DMSO control using one-sample t-tests in comparison to a hypothetical value of 0. (C) SP-C secretion was measured by ELISA. Shown are values normalized to CC without Pirfenidone. Significance was assessed using Wilcoxon matched pairs test. *N* = 6. (PDF 97 kb)

## Abbreviations

3D-LTCs: 3D ex vivo lung tissue cultures
Bleo: Bleomycin
CC: Control cocktail
Col: Collagen
ECM: Extracellular matrix
EMT: Epithelial to mesenchymal transition
FC: Fibrosis cocktail
FGF: Fibroblast growth factor
Fn: Fibronectin
Hopx: Homeodomain only protein X
IPF: Idiopathic pulmonary fibrosis
LPA: Lysophosphatidic acid
PCLS: precision cut lung slices
PDGF: Platelet-derived growth factor
Pdpn: Podoplanin
pmATII: primary murine alveolar epithelial type II
RXR: Retinoid X Receptor
S1P: Sphingosine 1-phosphate
SP-C: Pro surfactant protein C
TGF-β: transforming growth factor
TNF: Tumor necrosis factor
TTF-1: Thyroid transcription factor 1
VEGF: Vascular endothelial growth factor
WISP: Wnt1-inducible signaling protein
