# Supplementary figures and images for: Differential effects of Nintedanib and Pirfenidone on lung alveolar epithelial cell function in ex vivo murine and human lung tissue cultures of pulmonary fibrosis
Source: Respir Res. 2018 Sep 15;19:175. doi: 10.1186/s12931-018-0876-y (PMC6138909; doi:10.1186/s12931-018-0876-y)

Supplemental Fig S1

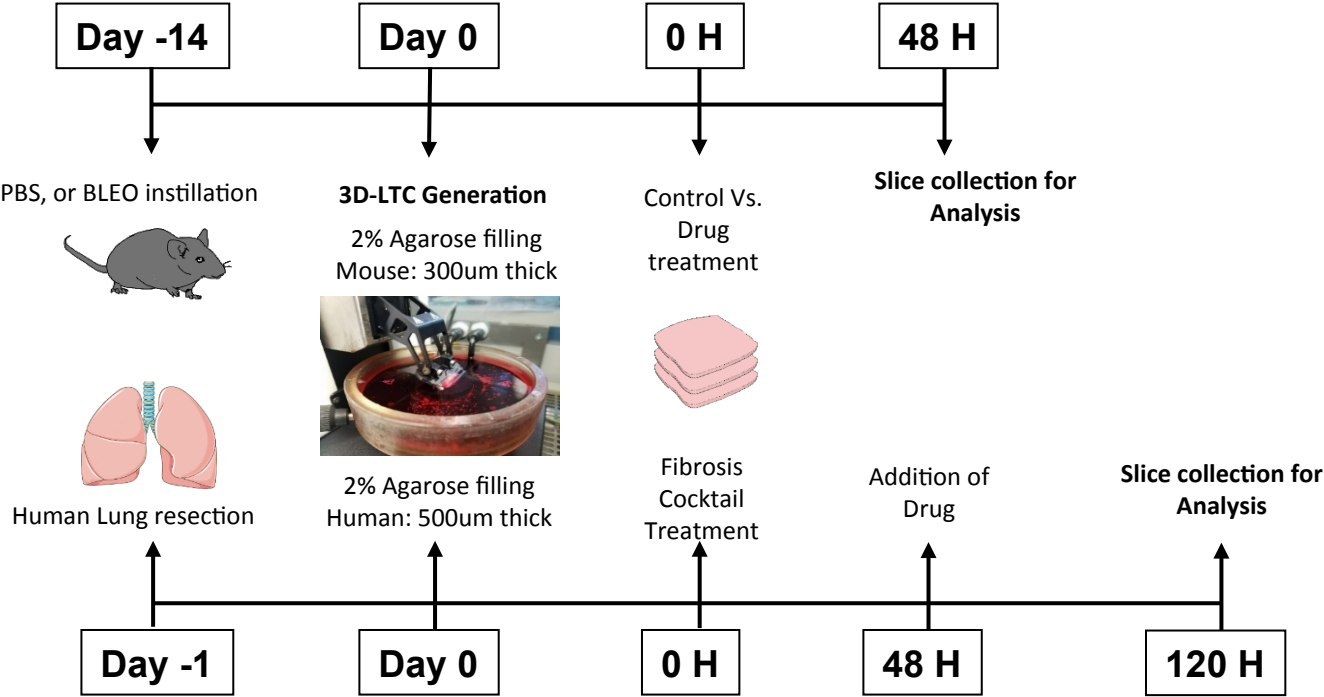

Supplement: Supplementary file 1 — Figure S1. Generation of mouse and human 3D-LTCs. Mouse lungs were harvested from PBS or Bleomycin treated mice or cancer resections from human patients were collected and filled with 2 or 3% low melting agarose, respectively. 300 or 500 μm thin 3D-LTCs were generated and cultured as indicated in each experiment. (PDF 90 kb) [file 12931_2018_876_MOESM1_ESM.pdf]

A

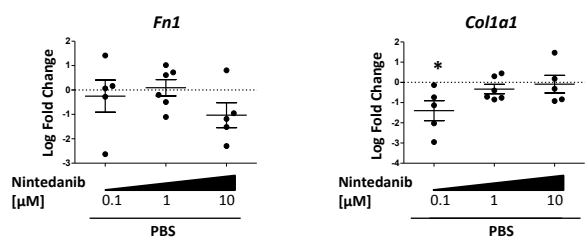

B

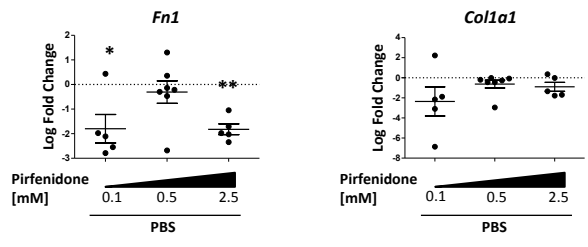

C

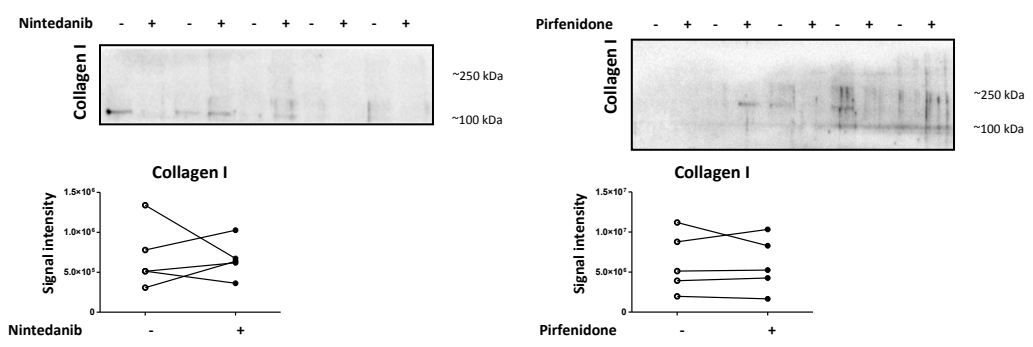

Supplement: Supplementary file 2 — Figure S2. Effect of Pirfenidone and Nintedanib on fibrotic marker in healthy 3D-LTCs ex vivo. Mice were instilled with PBS sacrificed at day 14. 3D-LTCs were generated and cultured for 48h. (A,B) The non-fibrotic 3D-LTCs were cultured for 48h in the presence of anti-fibrotic drugs (A) Nintedanib (0.1μM, 1μM, 10μM) (B) and Pirfenidone (100μM, 500μM, 2.5mM). Gene expression analysis by qPCR of fibrotic marker Fn1 and Col1a1. ΔΔCt relative to Hprt and respective DMSO control is presented as mean ± SEM, n = 5-7. Means were compared to respective DMSO control using one-sample t-tests in comparison to a hypothetical value of 0. (C) Collagen I secretion of nonfibrotic 3D-LTCs treated with Nintedanib (1μM) and Pirfenidone (500μM) was determined by WB and normalized to supernatant volume. n = 5. Significance: *p < 0.05, **p < 0.01. (PDF 166 kb) [file 12931_2018_876_MOESM2_ESM.pdf]

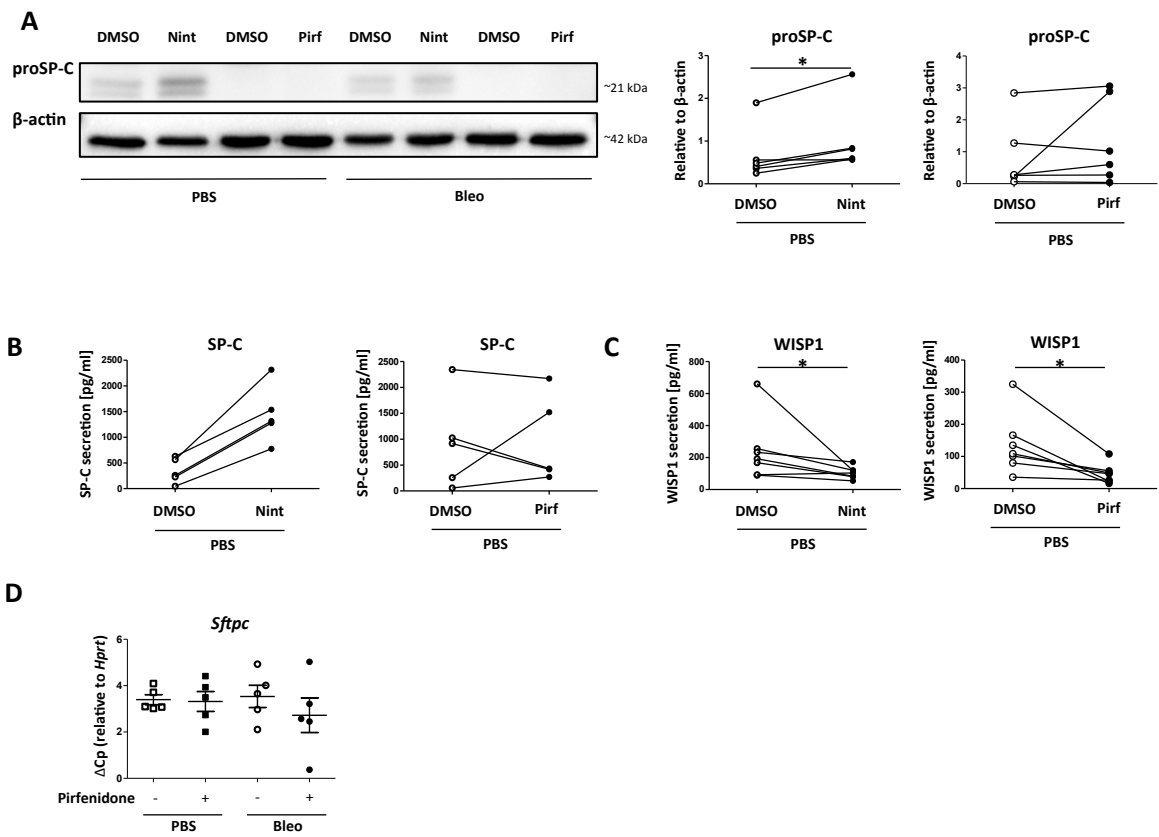

Supplement: Supplementary file 3 — Figure S3. Effect of Pirfenidone and Nintedanib on epithelial cell marker in non-fibrotic 3D-LTCs ex vivo (A-C) Fibrotic and non-fibrotic 3D-LTCs were cultured for 48h in the presence of anti-fibrotic drugs Nintedanib (1μM) and Pirfenidone (500μM). (A) proSP-C expression was assessed by Western blot. β-Actin was used as loading control. Quantification of proSP-C Western blot. Data was normalized to β-Actin. Significance was assessed using Wilcoxon matched pairs test, n = 6. (B, C) SP-C and WISP 1 secretion of non-fibrotic 3D-LTCs was determined by ELISA. Significance was assessed using Wilcoxon matched pairs test, n = 4-7. (D) Fibrotic and non-fibrotic 3D-LTCs were cultured for 48h in the presence of 2.5mM Pirfenidone. Gene expression analysis by qPCR of ATII marker Sftpc. ΔCt is presented as mean ± SEM, n = 5. Means were compared using repeated-measures one-way ANOVA followed by Newmann-Keuls post test. Significance: *p < 0.05. (PDF 748 kb) [file 12931_2018_876_MOESM3_ESM.pdf]

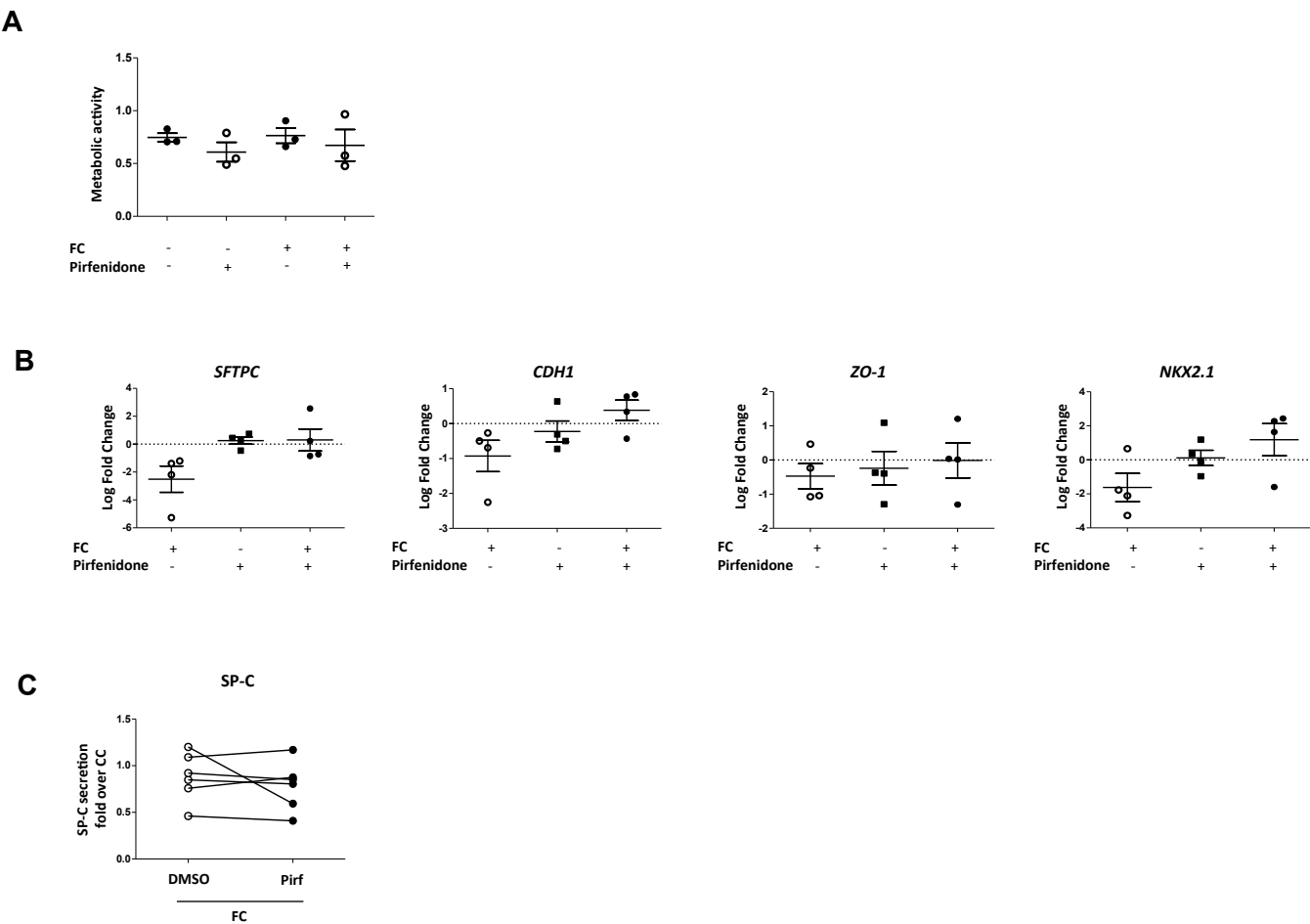

Supplement: Supplementary file 4 — Figure S4. Effect of Pirfenidone on ex vivo human PCLS model of IPF. (A-C) Punches were treated with CC/FC and Pirfenidone (500 μM) as indicated in (Fig. 4a). (A) Metabolic activity of punches 120h after treatment with CC/FC and cotreatment with Pirfenidone. N = 3. Significance was assessed by two-way ANOVA followed by Sidak’s multiple comparisons test. (B) Gene expression analysis by qPCR of epithelial cell marker SFTPC, NKX2.1, CDH-1, ZO-1. ΔΔCt is presented as mean ± SEM, n = 3. Means were compared to respective DMSO control using one-sample t-tests in comparison to a hypothetical value of 0. (C) SP-C secretion was measured by ELISA. Shown are values normalized to CC without Pirfenidone. Significance was assessed using Wilcoxon matched pairs test. N = 6. (PDF 97 kb) [file 12931_2018_876_MOESM4_ESM.pdf]
